# Lignin in Fibrous Feed as an Internal Digestibility and Transit Marker in Pigs

**DOI:** 10.1021/acsagscitech.4c00752

**Published:** 2025-04-24

**Authors:** Romy J. Veersma, Corentin Lannuzel, Walter J.J. Gerrits, Sonja de Vries, Gijs van Erven, Mirjam A. Kabel

**Affiliations:** a Laboratory of Food Chemistry, 4508 Wageningen University & Research, Bornse Weilanden 9, Wageningen 6708 WG, The Netherlands; b Animal Nutrition Group, Wageningen University & Research, De Elst 1, Wageningen 6708 WG, The Netherlands; c Wageningen Food & Biobased Research, Bornse Weilanden 9, Wageningen 6708 WG, The Netherlands

**Keywords:** internal marker, lignin, nutrient digestibility, mean retention time, pyrolysis-GC-MS, pigs

## Abstract

To assess the nutritional value of fibrous diets, external metal oxide-based markers are commonly used. Given the increasing restrictions on the use of these conventional markers, we investigated the inertness of lignin in pigs and correspondingly its potential as an internal marker. Hereto, feces and digesta were collected from pigs fed a diet containing wheat straw as sole lignin source, and lignin was quantified by ^13^C-IS py-GC-MS. Combined with detailed HSQC NMR and size-exclusion chromatography structural analysis, we revealed that lignin was recovered unmodified in feces. Comparison of lignin with TiO_2_ showed similar (*P* > 0.15) digestibility values for nitrogen (82.6 vs 80.2%) and fat (93.3 vs 92.5%), and a limited difference for dry matter (77.7 vs 74.1%, *P* = 0.01). Comparison of lignin with chromium-mordanted straw furthermore showed highly similar (*P* = 0.8) mean retention times of fibrous particles in the stomach. We thus confirm the potential of intrinsic lignin as a multifunctional marker for feed digestibility and digesta transit in pigs.

## Graphical abstract

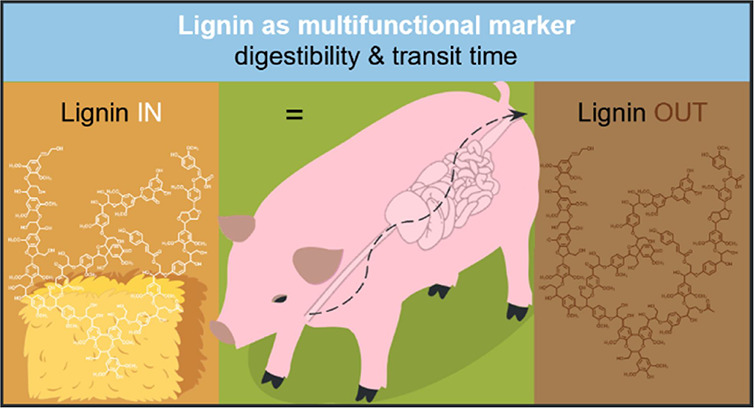

## 1 Introduction

Minimizing competition between resources for human food and animal feed unavoidably leads to increased inclusion levels of fibrous waste streams from the agrifood industry in animal feed.^1,2^ These high-fiber byproducts, mainly comprising plant cell wall material made up of the polysaccharides cellulose, hemicellulose, and the aromatic polymer lignin, may affect digestive processes and the nutritional value of the diets fed to the animals.^3,4^ To assess the latter, digestibility studies generally rely on external markers (or tracers). The ratio between marker(s) and nutrients in the feed and the digesta or feces is used to quantify net disappearance of nutrients, also referred to as apparent nutrient digestibility, and to monitor passage of feed throughout the gastrointestinal (GI-) tract.^5–9^

Selection of the appropriate marker(s) is crucial to method accuracy.^5,10^ The ideal marker is described as being inert, nontoxic, having negligible effects on digestion, fermentation, and digesta transit behavior, and flowing parallel with the nutrient(s) of interest (i.e., tracee).^5,7^ Common externally added markers to the animal’s diet, such as the metal oxides titanium­(IV)­oxide (TiO_2_) and chromium­(III)­oxide (Cr_2_O_3_), do not meet all requirements as they may separate from the tracee, especially in fibrous diets.^5,11^ Mordants as alternative external markers, in which the marker (e.g., chromium) is incorporated into high-fibrous particles, might reduce this separation problem, yet their preparation under severe conditions is laborious, hazardous, and selective.^5,12–14^ In addition, legislation for the use of conventional markers like TiO_2_ prohibits their use in commercial conditions in the European Union,^15^ which limits large-scale mapping of digestibility, urging the need for an alternative marker that is preferably intrinsically present in the feed.

Natural fiber-based markers that are an intrinsic part of the (fibrous) feed ingredient, seen as safe and not restricted in their use by legislation, may overcome the aforementioned marker-tracee separation problem.^5^ In that respect, the recalcitrant plant polymer lignin could be a promising internal marker. The recalcitrance of lignin is attributed to its heterogeneous structure, in which *p*-hydroxyphenyl (H), guaiacyl (G), and syringyl (S) units are incorporated in the aromatic lignin polymer, linked together by a variety of ether and carbon–carbon linkages.^16^ Still, the application of lignin as an inert marker in digestibility studies with monogastric animals, such as pigs, has been hampered because its true indigestibility has remained inconclusive. To the best of our knowledge, only a few fecal lignin recoveries in pigs have been reported, ranging from 58 to 110%.^17–20^ Expectedly, this broad recovery range relates to the applied gravimetric lignin quantification approaches (i.e., acid-detergent lignin and acid-insoluble (Klason) lignin), which are inherently prone to under- or overestimation of lignin contents because of their nonspecific nature.^21–23^

Recently, we have demonstrated that lignin can be specifically quantified in fecal samples from pigs by applying a novel, nongravimetrical lignin quantification method.^24^ This method is based on analytical pyrolysis coupled to gas chromatography with mass spectrometric detection (py-GC-MS), employing a uniformly ^13^C-labeled polymeric lignin isolate as internal standard (^13^C-IS).^25,26^ This ^13^C-IS py-GC-MS method requires simple sample preparation, i.e., water extraction, to overcome the effects of fecal matrix components on lignin quantification. Moreover, only limited amounts of material are needed for the sample preparation step (≤2 g) and subsequent analysis (±80 μg).^24^ Having this specific lignin quantification method in place allowed us, in this study, to investigate whether straw lignin added to the diet of pigs remains intact and unmodified throughout the GI-tract. Furthermore, it was hypothesized that wheat straw lignin functions well as a marker for nutrient digestibility and digesta transit time for the fibrous feed fraction. Therefore, in this study, 12 pigs were fed diets with wheat straw as sole fiber and lignin source, supplemented either as coarse (≤20 mm) or finely milled (≤1 mm) to a basal diet. The contrast in particle size of the wheat straw included in the diet was applied for passage rate determination in the stomach.^14^ Fecal samples were quantitatively collected over a 2-day collection period, and digesta samples were collected after a period of feeding the same diets with TiO_2_ and Cr-mordanted straw included as external markers. Collected material was analyzed for the contents of lignin and nutrients, and when relevant, for titanium and chromium contents.

Our two-step approach allows better insight into (1) the inertness of grass lignin in pigs and (2) the potential of lignin as an intrinsic marker to predict fibrous feed digestibility and digesta transit in pigs, compared to conventional metal oxide-based approaches.

## 2 Materials and Methods

### 2.1 Materials

All chemicals used were obtained from Sigma-Aldrich (St. Louis, MO, USA), Merck KGaA (Darmstadt, Germany), or VWR International B.V. (Amsterdam, The Netherlands). Water used in all laboratory experiments was purified by a Milli-Q water system (Millipore, Billerica, MA, USA). Wheat straw was obtained from Oldambt BV (Oostwold, The Netherlands) and coarsely chopped to ≤20 mm (WSC) or finely ground through a hammer mill, passing subsequently a 3 and 1 mm sieve (WSF) (Dinnissen BV, Sevenum, The Netherlands). Prior to analyses, WSC and WSF were milled through a 1 mm sieve at 12,000 rpm (ZM200; Retsch GmbH, Haan, Germany).^14^

Uniformly ^13^C-labeled (‘^13^C’; 97.7 atom % ^13^C) spring wheat plant (*Triticum aestivum* L. cv. “Baldus”), grown and provided by Isolife bv (Wageningen, The Netherlands), had previously been planetary ball-milled and sequentially water and dioxane extracted in our laboratory,^25^ to yield uniformly ^13^C-labeled wheat straw lignin isolate (89.5% w/w purity; ^13^C-WSL).

### 2.2 Collection of Pig Feces and Digesta Samples

Pig feces and digesta samples were obtained from our recent study,^14^ conducted at the research facilities of Wageningen University & Research (Wageningen, The Netherlands), and approved by the Dutch Central Committee of Animal Experiments (The Netherlands) under authorization number AVD1040020209705. In brief, 12 entire male pigs (51.6 ± 4.90 kg; Tempo x Topigs 20, Topigs Norsvin, Helvoirt, The Netherlands) were fed diets containing 150 g/kg wheat straw as sole fiber source, either supplied as WSC (*n* = 6) or WSF (*n* = 6), added to a basal diet (Table S1). On day 17, pigs were moved from groups (of three per pen) to individual metabolism pens, where total collection of feces was performed for 48 h. Feces were quantitatively collected by means of bags that were attached to rings glued around the anus of pigs. Feces collection was performed between 07:00–19:00. In total, a minimum of eight fecal samples were collected per pig and stored immediately at −20 °C pending further analyses. Prior to freeze-drying and milling (1 mm, see Section 2.1), collected feces were pooled per pig and subsampled. Feces samples obtained between 19:00 and 07:00 were weighed, contributing to the calculation of total fecal output, and subsequently discarded. Feed refusals were collected and recorded when present. On day 19, pigs were transferred back to their original pens. From day 20 onward, the markers Cobalt-EDTA (1 g/kg) to follow liquids and TiO_2_ (4 g/kg) to follow solids were included in the diet at the expense of maize starch (Table S1). Additionally, polyethylene glycol (5 g/kg) was included in the diets as an exploratory soluble marker in future studies and will not be further discussed. Chromium-mordanted wheat straw (either coarse or fine) replaced 15 g/kg of WSC or WSF as a marker for fibrous particles. From at least 36 h prior to dissections, pigs were fed meals every 6 h to approach steady-state passage of digesta in the large intestine. On the day of dissection (either day 25 or 26), pigs were fed once every hour to approach steady-state passage of digesta in the stomach and small intestine before euthanasia. Stomach (divided into proximal and distal), small intestine (length-based divided into three segments), and large intestine (length-based divided into four segments) contents were collected and weighed. Digesta samples were immediately stored at −20 °C and subsequently freeze-dried and milled (1 mm, see Section 2.1).

Unless stated otherwise, feed, feces, and digesta samples were further bead-milled (2 g, 50 mL stainless-steel jar, 2 φ15 mm stainless-steel balls, 2 min at 30 Hz) to a particle size of ≤ 250 μm (MM400, Retsch GmbH, Haan, Germany) prior to analysis.^14^

### 2.3 Preparation of Water-Unextractable Solids of Wheat Straw, Feces, and Digesta Samples to Allow Lignin Content Analysis

Water extraction of WSC, WSF, feces (FEC), distal stomach (DST), ileum (ILE), and rectum (REC) samples was performed according to Veersma et al.^24^ Briefly, 2 g of ≤ 1 mm milled material was dispersed in water (4.8% w/w) and extracted (1.5 h, 60 °C, intermittent shaking). After removal of water extractives by centrifugation (4600 × *g*, 5 min, 20 °C), the residue was washed with 35 g of water. After removal of the supernatant, the water-unextractable solids (WUS) material was freeze-dried and bead-milled (Section 2.2; MM400) to obtain WSC_WUS_, WSF_WUS_, and per pig FEC_WUS_, REC_WUS_, ILE_WUS_, and DST_WUS_ samples, which were subjected to ^13^C-IS py-GC-MS analysis.

### 2.4 Mild Organosolv Lignin Isolation of Wheat Straw and Feces

FEC_WUS_ of six pigs fed with the WSC-diet was pooled in equal mass amounts (FEC_P‑WSC_). Mild organosolv fractionation was performed on WSC_WUS_ and FEC_P‑WSC_ according to van Erven et al.^27^ In brief, 8 mL of solvent (80% w/w γ-valerolactone, 19% w/w water, and 1% w/w H_2_SO_4_) was added to a 500 mg sample and thoroughly vortexed before being added to a Stuart SBH200*D*/3 heating block (Cole Palmer, Vernon Hills, IL, USA) at 120 °C. The mixtures were heated for 30 min, with vortexing every 5 min. After the extraction, samples were cooled on ice and centrifuged (2500 × *g*, 2 min, 20 °C) to separate soluble and insoluble fractions. Then, 6 mL of the supernatant was transferred to 40 mL of water, vigorously mixed, and left to precipitate at 4 °C for 24 h. Precipitates were obtained by centrifugation (4700 × *g*, 2 min, 20 °C), washed twice with 25 mL of water set to pH 2, and air-dried at room temperature to yield WSC_LIG_ and FEC_P‑WSC‑LIG_, for alkaline size-exclusion chromatography (SEC) and heteronuclear single quantum coherence (HSQC) NMR analysis.

### 2.5 Quantitative py-GC-MS with ^13^C-Wheat Straw Lignin as Internal Standard

Lignin content and structural analyses of samples mentioned in Section 2.3 from all 12 pigs were performed as previously described by Veersma et al.,^24^ based on van Erven et al.^26^ To each accurately weighed sample (80 μg; XP6 excellence-plus microbalance, Mettler Toledo, Columbus, OH, USA), 10 μL of ^13^C-WSL (1 mg/mL 50:50 chloroform:ethanol) was added as an internal standard (IS). Lignin contents and relative abundances of lignin-derived pyrolysis products (Table S2) were calculated as described previously.^26^

Additionally, the lignin content of WSC (mentioned in Section 2.1) in the presence of titanium dioxide (TiO_2_) or sodium dichromate dihydrate was determined to assess the possible interference of these metal oxides with our lignin quantification method. Hereto, WSC was homogeneously mixed with TiO_2_ or sodium dichromate dihydrate (2 or 6% (w/w)), weighed, and analyzed as described above.

### 2.6 Dry Matter, Ash, Fat, Protein, Starch, and Nonstarch Polysaccharide Analysis

Dry matter, ash, and fat content of (nonextracted) ≤1 mm milled samples (WSC, WSF, and per pig FEC, REC, ILE, and DST) were determined according to ISO 6496 (1999),^28^ ISO 5984 (2002),^29^ and ISO 6492 (1999).^30^ Protein and starch content of bead-milled samples were determined according to AOAC 990.03 ^31^ and AOAC 996.11,^32^ with some modifications as described in detail by Veersma et al.^24^ Preparation of nonstarch polysaccharides (NSP), corresponding neutral sugar composition analysis and quantification, as well as quantification of uronic acid, was performed on bead-milled samples according to Jonathan et al.,^33^ Englyst et al.,^34^ Englyst & Cummings,^35^ Blumenkrantz & Asboe-Hansen,^36^ and Thibault & Robin,^37^ with few modifications as previously described by Veersma et al.^24^

### 2.7 Marker Analysis by Inductively Coupled Plasma Optical Emission Spectrometry (ICP-OES)

Titanium (Ti) and chromium (Cr) concentrations of (nonextracted) ≤1 mm milled samples (per pig REC, ILE, and DST) were determined using inductively coupled plasma optical emission spectrometry (ICP-OES) after ashing and microwave digestion according to Lannuzel et al.^14^ Ti concentration in feed was calculated using the dosed Ti content, as verified by the analyzed Ti content in the diets. Cr concentration in feed was calculated using the analyzed Cr content in the Cr-mordanted wheat straw.

### 2.8 Alkaline Size Exclusion Chromatography (SEC)

WSC_LIG_ and FEC_P‑WSC‑LIG_ lignin isolates were analyzed for molecular weight distribution by alkaline SEC as described by Constant et al. (method D).^38^ Briefly, samples were dissolved in 0.5 M NaOH (eluent) at a concentration of 1 mg mL^–1^, and separation was performed by using two TSKgel GMPWxl columns (7.8 × 300 mm, particle size 13 μm) in series, equipped with a PWxl guard column (6.0 × 40 mm, particle size 12 μm). Absorption was monitored at 280 nm with an ultraviolet spectroscopy detector. Sodium polystyrenesulfonate (PSS) standards and phenol were used for calibration. Protobind 1000 lignin (GreenValue SA, Switzerland) was used as a reference.

### 2.9 Nuclear Magnetic Resonance Spectroscopy (NMR)

^1^H–^13^C HSQC measurements were performed on a Bruker AVANCE III 400 MHz instrument equipped with a 5 mm BBI probe with a z-gradient (5 G cm–1 A–1). Approximately 20 mg of WSC_LIG_ or FEC_P‑WSC‑LIG_ was dissolved in 0.6 mL of DMSO-*d*_6_. Spectra were recorded by using the adiabatic “hsqcetgpsisp2.2” pulse sequence using the following parameters: a spectral width of 4800 Hz (12 ppm) in F1 (^1^H) using 1922 increments for an acquisition time (AQ) of 0.2 s and an interscan delay (D1) of 1.0 s and a spectral width of 20,000 Hz (200 ppm) in F2 (^13^C) using 322 increments with an AQ of 8 ms with 32 scans per increment. The ^1^JCH used was 145 Hz. Processing used Gaussian apodization (GB = 0.001, LB = −0.2) in ^1^H and a squared cosine function in ^13^C. The central solvent peak was used as an internal reference (δ_C_ = 39.5 ppm; δ_H_ = 2.49 ppm). The spectra were processed using TopSpin 4.0 software (Bruker, Billerica, MA, US). Semiquantitative analysis of the volume integrals was performed according to del Río et al.^39^ with slight modifications as previously described.^27^

### 2.10 Calculations for Lignin Recovery, Nutrient Digestibility, and Mean Retention Time

#### 2.10.1 Recovery of Lignin in Feces

Over the 2 day total feces collection period (Section 2.2), the feed intake and the fecal output were recorded per pig. From the inclusion level in the feed, the wheat straw intake per pig was calculated. Since wheat straw was the only source of lignin in the diets of the pigs, the amount of lignin was quantified in WSC and WSF with ^13^C-IS py-GC-MS (Section 2.5), to determine the lignin input per pig. Similarly, lignin in feces was quantified to calculate the lignin output per pig. The fecal lignin recovery was calculated using eq 1:

$$\mathrm{null}\;\mathrm{lignin}\;\mathrm{recovery}\;(\%)=(\frac{\mathrm{lignin}\;\mathrm{output}}{\mathrm{lignin}\;\mathrm{intake}})\times 100$$ 1

where lignin intake and lignin output are expressed in grams of dry matter.

#### 2.10.2 Apparent Rectal and Ileal Digestibility of Nutrients

The apparent rectal and ileal digestibility of nutrient *X*, respectively ARD and AID, was calculated using eq 2:^7^

$$\mathrm{ARD}\;\mathrm{or}\;\mathrm{AID}\;(\%)=(1-\frac{M_{\mathrm{feed}}\times X_{\mathrm{digesta}}}{M_{\mathrm{digesta}}\times X_{\mathrm{feed}}})\times 100$$ 2

where *M*_feed_, *M*_sample_, *X*_feed_, and *X*_sample_ (g/kg) are concentrations of the marker (*M*) and nutrient (*X*) in the feed and in the digesta (rectum or ileum). The marker was either lignin or titanium. Note that due to the absence of Ti in the feed during the total feces collection (Table S1), corresponding apparent total tract digestibility values could not be determined or compared.

#### 2.10.3 Mean Retention Time (MRT) of Fibrous Feed

The mean retention time (MRT) of fibrous feed particles in the distal stomach was calculated using eq 3,^5^ assuming that steady-state passage of digesta was reached:

$$\mathrm{MRT}\;(h)=(\frac{M_{\mathrm{DST}}\times Q_{\mathrm{DST}}}{M_{\mathrm{feed}}\times F_{\mathrm{intake}}})$$ 3

where *M*_feed_ and *M*_DST_ (g/kg) are concentrations of the marker (*M*) in the feed and in the distal stomach (DST) sample, *Q*_DST_ (g) is the marker pool size, and *F*_intake_ is the feed intake (g/h) prior to dissection. Marker was lignin or chromium (from mordanted straw) to follow fibrous particles.

### 2.11 Statistical Tests

Analytical and biological triplicates or duplicates were averaged, and standard deviations were calculated with Microsoft Excel (STDEV.S). Pig was the experimental unit for statistical analyses. Estimated digestibility values were compared using a general linear mixed model (PROC MIXED, SAS version 9.4, SAS Institute Inc., Cary, NC), with method (lignin vs titanium), segment (ileum vs rectum), diet (WSC, WSF), and their interactions as fixed effects. Preliminary analysis revealed no diet interactions (diet × method, diet × segment, and diet × method × segment; *P* > 0.3); hence, these interactions were omitted from the final model. Estimated MRT in the distal stomach was compared using a general linear mixed model with method (lignin vs chromium), diet (WSC, WSF), and their interactions as fixed effects. For both models, the method was modeled as a within-subjects random (R-side) effect, assuming a compound symmetry covariance structure, to account for repeated observations (ileum and rectum samples originating from the same pig) for the two methods within the pig. For the sake of brevity, only the main effects of the methods are presented. Model assumptions and the goodness of fit of models were evaluated through the distribution of conditional Pearson residuals, the null model likelihood ratio test, and Akaike and Bayesian information criteria. The relation between AID, ARD, and MRT estimated with lignin versus titanium or chromium as marker was further evaluated by simple linear regression (Microsoft Excel) using the model *y* = *a* + β*x*, where *y* is AID, ARD, or MRT estimated with lignin, *a* is the intercept, β is the slope, and *x* is AID, ARD, or MRT estimated with titanium (AID, ARD) or chromium (MRT). When applicable, Student *t-*tests (TTEST, two-sample unequal variance) were performed in Excel in order to express significance by a two-tailed *P*-value. Data are presented as raw means plus standard deviation, unless indicated otherwise. Differences among means with *P* < 0.05 were accepted as statistically significant.

## 3 Results and Discussion

### 3.1 Lignin Is Largely Inert during Passage through the Pig’s Digestive Tract

The aim of this research was to evaluate the potential of the aromatic plant polymer lignin to serve as a marker in digestibility studies. Hereto, a prerequisite is that the marker, i.e., lignin, is inert in the GI-tract. The indigestibility of lignin was assessed in this study with ^13^C-IS py-GC-MS, both by quantifying and comparing structural features of lignin in the feed and pig feces, obtained from a controlled period of total fecal collection.^14^ In the feed, wheat straw was the only source of lignin present and included either as coarsely chopped (WSC) or finely ground (WSF) material (Table S1). As previously validated,^24^ water extraction considerably improved straw lignin quantification by ^13^C-IS py-GC-MS, particularly in fecal (FEC) samples. Hence, lignin in WSC, WSF, and corresponding FEC samples were quantified in their respective WSC_WUS_, WSF_WUS_, and FEC_WUS_ samples of 12 pigs (Table S3). Determined lignin contents in these WUS samples were subsequently expressed as lignin contents of nonextracted samples by correcting for the extraction recovery (Tables S4 and S5), and for FEC averaged (*n* = 6) per diet, as presented in Table 1.

**Table 1:** Lignin Content (% w/w, Dry Matter) of WSC, FEC_WSC_ (*n* = 6; Table S4), WSF and FEC_WSF_ (*n* = 6; Table S5), as Determined with ^13^C-IS Py-GC-MS, Expressed in Nonextracted Samples^d^

	**WSC**	**FEC**_**WSC**_	**WSF**	**FEC**_**WSF**_
lignin content (% w/w)	18.2 ± 0.4	13.7 ± 0.6	17.5 ± 0.4	14.6 ± 0.7
lignin recovery (diet to feces) (%)		69 ± 6		73 ± 11
lignin subunits (%)				
H	12.3 ± 0.4	14.1 ± 0.5	13.5 ± 0.0	14.2 ± 0.0
G	59.6 ± 2.2	59.6 ± 0.5	59.5 ± 0.0	59.5 ± 0.0
S	28.1 ± 1.8	26.3 ± 0.6	27.0 ± 0.0	26.3 ± 0.0
S/G	0.47 ± 0.0	0.44 ± 0.0	0.45 ± 0.0	0.44 ± 0.0
coumaryl, coniferyl, and sinapyl alcohol (%)				
*t*-coumaryl alcohol (CouA)	2.8 ± 0.1	3.1 ± 0.1	2.9 ± 0.0	2.9 ± 0.1
*t*-coniferyl alcohol (ConA)	57.8 ± 2.7	58.1 ± 0.6	57.7 ± 1.3	57.0 ± 0.8
*t*-sinapyl alcohol (SinA)	39.4 ± 2.6	38.8 ± 0.5	39.4 ± 1.3	40.1 ± 0.8
SinA/ConA	0.68 ± 0.1	0.67 ± 0.0	0.68 ± 0.0	0.70 ± 0.0
lignin structural moieties (%)^a^				
unsubstituted	6.5 ± 0.2	6.9 ± 0.2	6.5 ± 0.0	6.7 ± 0.0
methyl	4.1 ± 0.3	4.3 ± 0.1	4.3 ± 0.0	4.2 ± 0.0
vinyl	41.1 ± 1.2	42.8 ± 1.5	43.8 ± 0.0	44.4 ± 0.0
4-vinylphenol	8.9 ± 0.4	10.1 ± 0.4	10.1 ± 0.0	10.4 ± 0.0
4-vinylguaiacol	27.3 ± 1.6	28.5 ± 1.1	28.9 ± 0.0	29.7 ± 0.0
Cα-ox	4.2 ± 0.3	4.3 ± 0.1	3.9 ± 0.0	4.2 ± 0.0
Cβ-ox	1.1 ± 0.1	1.1 ± 0.0	1.0 ± 0.0	1.1 ± 0.0
Cγ-ox	38.9 ± 1.3	35.5 ± 1.5	35.9 ± 0.0	34.3 ± 0.0
miscellaneous	4.1 ± 0.0	5.1 ± 0.2	4.5 ± 0.0	5.0 ± 0.0
PhCγ^b^	43.9 ± 1.5	41.5 ± 1.6	41.3 ± 0.0	40.4 ± 0.0
PhCγ-corrected^c^	43.3 ± 1.4	40.9 ± 1.6	40.6 ± 0.0	39.7 ± 0.0

a Some lignin-derived pyrolysis products can be indicative for more than one structural moiety, hence the total exceeds 100%.

b Phenols with intact α,β,γ carbon side chain.

c Phenols with intact α,β,γ carbon side chain, excluding diketones and vinylketones.

d Corresponding lignin recoveries (%) from the diet to feces were calculated based on the averaged total lignin input and output (Table S6). ^13^C-IS py-GC-MS relative abundances of lignin compounds in WSC, FEC_WSC_, WSF, and FEC_WSF_ were corrected for relative response factors and the relative abundance of ^13^C analogs. Sum based on structural classification according to Table S2. Means ± standard deviation of analytical triplicates (WSC; WSF) and biological replicates (FEC).

During the 48 h of total collection of feces, total dry matter intake and fecal output per individual pig were recorded. Combining these data with the lignin contents allowed the calculation of total lignin intake and output per pig, i.e., a lignin mass balance (Table 1; Table S6). Following this, 68.8% ± 5.6 and 73.0% ± 11.3 of lignin were recovered in feces of pigs fed with a WSC- or a WSF-based diet, respectively.

To the best of our knowledge, this is the first study that shows lignin recoveries in (pig) feces based on a lignin-specific ^13^C-IS py-GC-MS quantification method. To date, merely gravimetric lignin quantification methods such as acid-detergent lignin (ADL) and acid-insoluble (Klason) lignin have been used, resulting in a broad range of fecal lignin recoveries published for pigs (58–110%).^17–20^ As stated previously, these gravimetric methods lack specificity and are therefore highly prone to under- or overestimating lignin contents,^21–23^ especially in complex sample matrices such as feces.^24^

Not only was lignin recovered rather well (±70%), but the structural features of recovered fecal lignin were highly similar to the wheat straw lignin structure in the feed, both in terms of subunit composition and detailed structural moieties (Table 1). Additionally, the abundance of the hydroxycinnamates ferulic acid and *p*-coumaric acid, represented by the pyrolysis products 4-vinylguaiacol and 4-vinylphenol, respectively, remained virtually unchanged (Table 1). The minor increase in fecal *p*-hydroxyphenyl (H−) units might be related to other nonlignin cell wall components (e.g., aromatic amino acids in protein),^25,40^ also resulting in H-unit pyrolysis products. Indeed, semiquantification of indole by py-GC-MS in water-extracted straw and feces samples revealed the presence of such proteinaceous compounds, particularly in the feces samples (Figure S1). The strong similarity in distribution profile of the lignin-specific 4-hydroxyphenylpropanoid pyrolysis products (coumaryl alcohol, coniferyl alcohol, and sinapyl alcohol) in straw and feces (Table 1) further supported the inertness of the lignin structure.

To further substantiate this inertness of the lignin structure, lignin was mildly isolated from water-extracted coarse straw and corresponding pooled feces samples to allow more detailed structural analysis. In accordance with ^13^C-IS py-GC-MS lignin structural analysis, HSQC NMR analysis of the respective WSC_LIG_ and FEC_P‑WSC‑LIG_ lignin isolates demonstrated very similar structural profiles (Figure 1). Semiquantitative analysis of the volume integrals established a similar subunit composition, lignin interunit abundance and composition, and abundance of the hydroxycinnamates ferulate and *p*-coumarate and the flavonoid tricin (Table 2).

**Figure 1:**
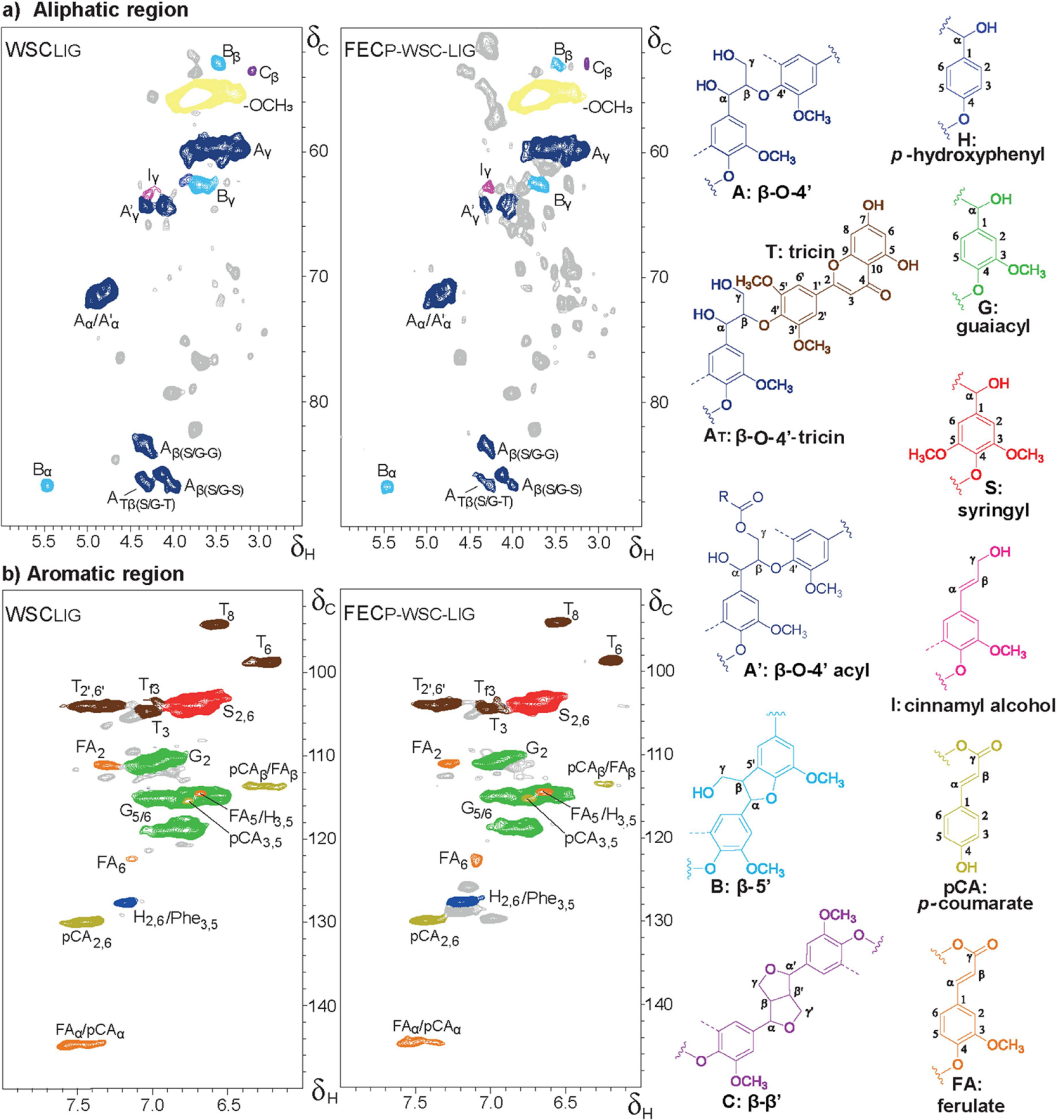
(a) Aliphatic and (b) aromatic regions of ^1^H–^13^C HSQC NMR spectra of lignin isolated from WSC (WSC_LIG_) and FEC_p‑WSC_ (FEC_p‑WSC‑LIG_). Unassigned signals are presented in gray. Dotted lines in depicted structures represent a hydroxy or methoxy group; wavy lines indicate the main positions for further coupling.

**Table 2:** Semiquantitative ^1^H–^13^C HSQC NMR Structural Characterization of WSC_LIG_ and FEC_P‑WSC‑LIG_ ^a^

		**WSC**_**LIG**_	**FEC**_**P‑WSC‑LIG**_
HSQC NMR	lignin subunits (%)		
	H	3.1	1.6
	G	61.1	63.8
	G_ox_	0.7	0.0
	S	34.2	34.6
	S_ox_	0.9	0.0
	S/G	0.57	0.54
	lignin interunit linkages (per 100 Ar)		
	β-*O*-4 aryl ether	43.2	49.1
	β-5 phenylcoumaran	5.1	6.3
	β-β resinol	0.9	0.0
	hydroxycinnamates (per 100 Ar)		
	ferulate	5.1	5.9
	*p*-coumarate	8.8	8.7
	flavonoids (per 100 Ar)		
	tricin	16.7	15.6
SEC	total elution profile		
	*M*_w_ (g/mol)	2860	4225
	*M*_n_ (g/mol)	550	610
	Đ (*M*_w_/*M*_n_)	5.2	6.9

a Alkaline SEC molecular weight (*M*_w_) distribution of lignin isolates was calculated based on PSS standards. Ar = aromatic rings.

The slight increase in β-*O*-4 aryl ether linkages of the isolated fecal lignin (Table 2) was not observed for nonisolated fecal lignin (Table 1; PhCγ products, indicative for intact β-*O*-4 linkages^41^), hence suggesting that the isolated fecal lignin population might not be fully representative of the complete lignin structure in FEC. However, and most importantly, with both lignin structural analysis methods, we confirmed that there was no indication of wheat straw lignin modification nor depolymerization in the pig’s GI-tract. This notion was corroborated from the molecular weight distribution perspective by SEC analysis showing similar elution profiles (Figure S2). The higher apparent molecular weight (*M*_w_) and dispersity (*Đ*) observed for lignin isolated from FEC_P‑WSC_, as compared to WSC (Table 2), were likely due to the presence of covalently bound carbohydrates that were coextracted, rather than due to the presence of an actual higher molecular weight fraction. Indeed, the aliphatic region of the HSQC spectrum of this isolate showed more abundant carbohydrate-derived signals (Figure 1; FEC_P‑WSC‑LIG_). Nonetheless, the conclusion remains unchanged, i.e., no substantial changes were observed in the structural features of fecal lignin compared to the lignin in the wheat straw that was fed to the pigs.

### 3.2 Use of Lignin as a Marker Yields Sound Nutrient Digestibility Estimates

Having established that lignin remained structurally inert during GI passage, we evaluated its potential as a marker to estimate nutrient digestibility against conventional markers. In the current setup of the animal experiment, the total feces collection period was performed prior to the inclusion of conventional markers in the diets (Table S1). This setup was chosen to ensure the absence of any possible effects of these external markers on the quantification of lignin in feed and feces with ^13^C-IS py-GC-MS and associated lignin recovery. Consequently, fecal recoveries of these conventional markers, including titanium from TiO_2_, were not available for this study.

For a fair comparison between markers, it is important to note that, as described in Section 3.1 for lignin, conventional markers also range in their fecal recoveries published. The reported recovery of titanium in pig feces ranged between 76 and 111% and of chromium between 73 and 96%.^20,42–46^ These rather broad ranges in fecal recovery of the supposedly inert external markers are speculated to result, among others, from analytical recovery of markers,^5^ sample handling and representability, and conditions of the total collection of feces (e.g., adaptation time, duration of the collection period, amount of samples).^42,47,48^ The ideal marker is often described as being truly inert and should in theory therefore be fully recovered;^20^ however, this seems unrealistic given previously described incomplete titanium, chromium, and lignin recoveries. Nevertheless, the ± 70% lignin recovered in feces in our study was fairly constant among individual pigs (Tables S4 and S5), regardless of the diet, and the lignin structure was unaffected by the GI-tract of the pigs.

After the period of total collection of feces, the pigs were fed the same wheat-straw-based diet, with added external markers including TiO_2_ (Table S1). Digesta (spot) samples were obtained from rectum (REC) and ileum (ILE), and analyzed for lignin and titanium contents, after having established that lignin quantification by our ^13^C-IS py-GC-MS methodology was not affected by the examined titanium (or chromium) concentrations (Figure S3).

To assess the potential of lignin as a nutrient digestibility marker for fibrous feed, apparent (rectal and ileal) digestibilities of dry matter, nitrogen, and fat were calculated and compared with titanium or lignin as a marker (Figure 2; Table S7). In the present study, wheat straw (135 g/kg) was included in the diets in coarse or fine form. Preliminary statistical evaluation indicated that there was no effect of diet (i.e., WSC or WSF; *P* > 0.3) on the lignin or titanium marker method (see also Section 2.11). In other words, the relation between nutrient digestibility coefficients determined with lignin or titanium as a marker was unaffected by the straw particle size in the diet fed to the pigs. Similarly, the aforesaid relation was not affected by segment (i.e., rectum or ileum; *P* > 0.1). Due to the absence of a diet or segment effect, data were pooled for statistical analyses. Employing intrinsic feed lignin as a marker yielded similar mean digestibility estimates as titanium (*P* > 0.15) for nitrogen (82.6% vs 80.2%) and fat (93.3% vs 92.5%) (Figure 2). The mean dry matter (DM) digestibility value determined with lignin was 3.6%-units greater (*P* = 0.01; 77.7% vs 74.1%) compared to titanium.

**Figure 2:**
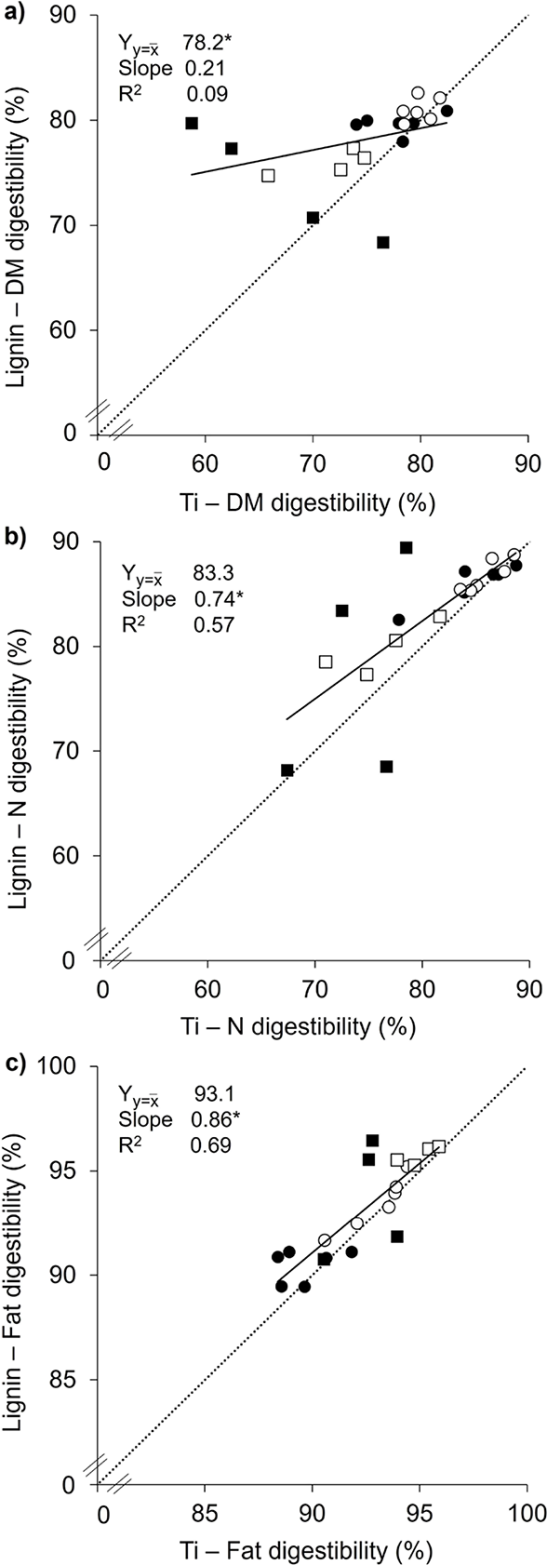
Apparent rectal digestibility (ARD; *n* = 12) and apparent ileal digestibility (AID; *n* = 8) of (a) dry matter (DM), (b) nitrogen (N) and (c) fat, estimated with titanium (Ti) as marker compared to lignin as marker. circle solid = rectum samples of WSC-fed pigs, circle open = rectum samples of WSF-fed pigs, box solid = ileum samples of WSC-fed pigs, box open = ileum samples of WSF-fed pigs. Data are presented as individual observations and regression lines with confidence limits and the corresponding regression parameters. Dotted lines represent *y* = *x*. *Y*_*y*=*x̅*_ = *y* at *x̅*, *R* ^2^ = coefficient of determination adjusted for the number of terms. Asterisks indicate that *Y*_*y*=*x̅*_ is significantly different from *x̅* or slope is significantly different from 0 (*P* < 0.05).

The two distinct outliers, showing a 15–21% unit higher (ileal) DM digestibility value calculated with lignin as a marker (Figure 2a), were not similarly observed for nitrogen (N) and fat digestibility (Figure 2b,c). The formula for the calculation of apparent digestibility values of a specific nutrient (eq 2, see Section 2.10.2) is dependent on both the ratio marker_digesta_/marker_feed_ and the ratio nutrient_digesta_/nutrient_feed_. Further examination of the two aforementioned outliers revealed that the ratio between lignin_digesta_/lignin_feed_ and Ti_digesta_/Ti_feed_ for these two samples is clearly increased compared to the other digesta samples (Table S8). Moreover, the ratio DM_digesta_/DM_feed_ is much less variable (1.0 ± 0.0) between the digesta samples than the ratio N_digesta_/N_feed_ or Fat_digesta_/Fat_feed_ (Table S8). It can therefore be postulated that the increased lignin_digesta_/lignin_feed_ versus Ti_digesta_/Ti_feed_ caused a more pronounced effect on, particularly, ileal DM digestibility values. Interestingly, lignin_digesta_/lignin_feed_ was similar to Ti_digesta_/Ti_feed_ (*P* = 0.9), further attributing to the competence of lignin as a marker.

The above-described comparative results demonstrated that digestibility values of dietary nutrients between the use of either lignin or titanium as a marker were in good agreement, hence substantiating the potential of intrinsic feed lignin as a digestion marker in (large-scale) digestibility studies for pigs. This conclusion is in line with the study of Jagger et al.,^20^ where apparent ileal and fecal digestibility of nitrogen in pigs determined by ADL lignin and TiO_2_ agreed well. To the best of our knowledge, no further studies have to date been conducted where the use of intrinsic lignin as a marker in pig studies was evaluated against titanium as an external marker. Likely, incomplete (<100%) fecal lignin recoveries have thus far critically impeded the application of this plant polymer as an internal marker for the prediction of feed digestibility.^49^ However, as previously discussed, it is important to recognize that conventional markers are also generally not fully recovered in feces. Therefore, striving for 100% recovery of a digestibility marker might not be realistic.

### 3.3 Mean Retention Times of Fibrous Particles Correspond Well when Using Lignin or Chromium as a Marker

In addition to nutrient digestibility values, markers can also provide valuable insight into digesta transit.^5,13,14^ For instance, a chromium-mordanted feed ingredient (e.g., straw, bran, or hull) is added to the diet as a marker for (insoluble) fibrous particles.^5^ However, these mordants are laborious to prepare,^12^ are associated with safety risks,^50^ while the mordanting procedure results in (partial) modification of the feed ingredient.^51^ As intrinsically present in the feed, lignin is intertwined with other plant cell wall fibers (e.g., cellulose, xylan).^4^ Hence, it was hypothesized that lignin in fibrous feed can also function as a marker to study the transit of fibrous feed particles. Accordingly, we determined the mean retention time (MRT) of fibrous particles in the stomach of pigs (DST; *n* = 12), using lignin or chromium (mordant) contents (Figure 3). Regardless of the diet (*P* = 0.6), comparable MRT values (*P* = 0.8) were observed between lignin (3.9 h ± 2.9) and chromium (3.7 h ± 2.6) over a considerable MRT range.

**Figure 3:**
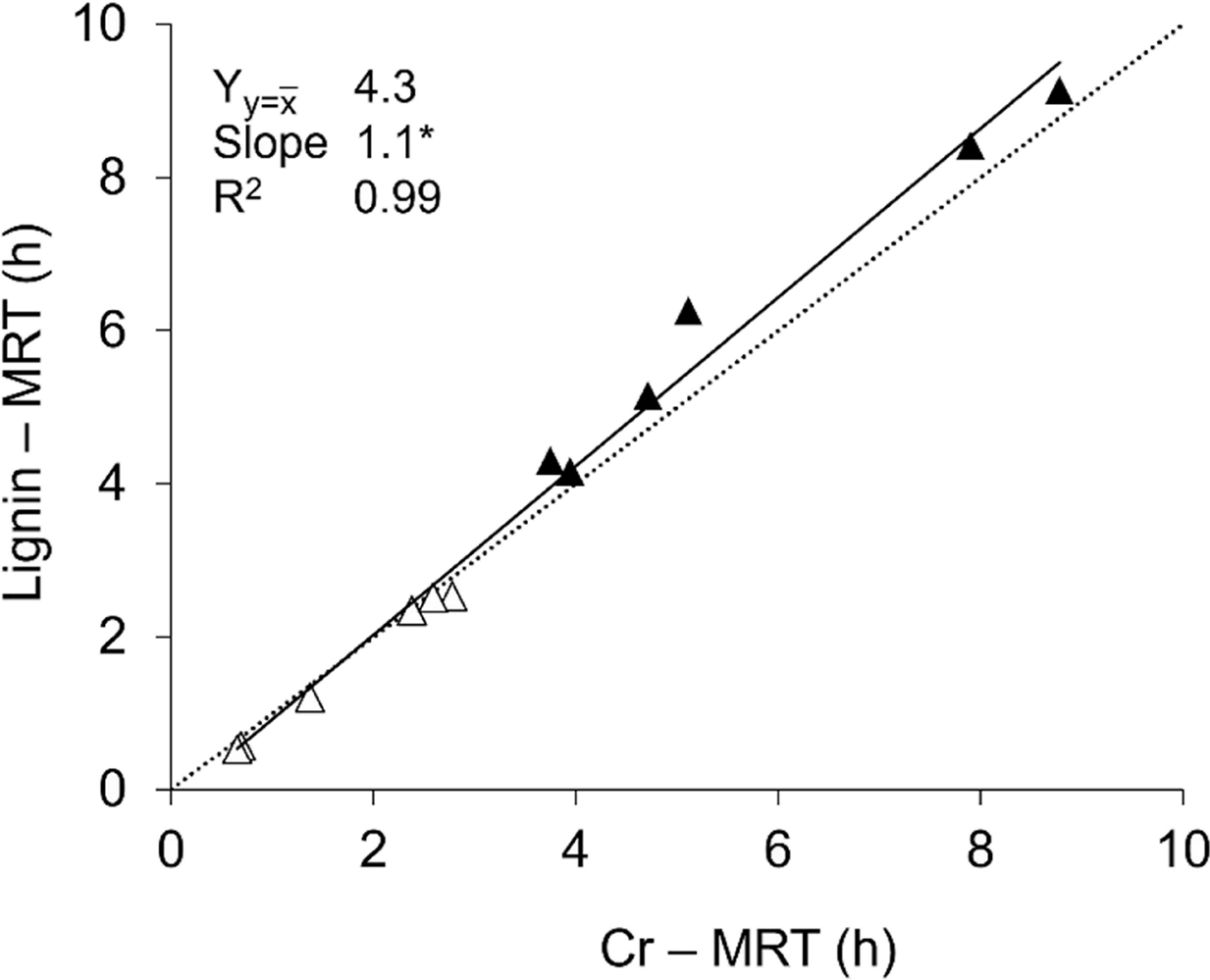
Mean retention time (MRT) of fibrous particles (*n* = 12), estimated with chromium (Cr) as marker compared to lignin as marker. triangle up solid = distal stomach samples of WSC-fed pigs; triangle up open = distal stomach samples of WSF-fed pigs. Data are presented as individual observations and regression lines with confidence limits and corresponding regression parameters. The dotted line represents *y* = *x*. Y_*y*=*x̅*_ = *y* at *x̅*, *R* ^2^ = coefficient of determination adjusted for the number of terms. Asterisks indicate the *Y*_*y*=*x̅*_ is significantly different from *x̅* or slope is significantly different from 0 (*P* < 0.05).

The distinct shortening effect of straw particle size reduction on MRT of fibrous particles in the stomach of pigs (based on Cr-mordants), as previously reported by Lannuzel et al.,^14^ was also revealed when employing feed lignin as a marker (Figure 3). Our findings demonstrate that intrinsic lignin functions well as both a marker for nutrient digestibility (Section 3.2) and transit time of fibrous feed. Compared with TiO_2_, other metal oxides, and (Cr-)­mordanted fibers, the use of lignin as internal digestibility and transit marker provides several advantages: (I) lignin-specific quantification method in place, with moderately high-throughput and simple sample preparation, (II) forms integral part of the (fibrous) feed, (III) no legislative restrictions for use in animal nutrition and (large-scale) studies, and (IV) safe to use,^15^ both during feed production and consumption by animals. Still, further studies are warranted, in particular, whether or at what inclusion levels lignin possibly affects the digestibility of other feed components. Hence, lignin, as a multifunctional and internal marker, now calls for further validation and implementation in future high-fiber feed digestibility studies.

## Supplementary Material



## Data Availability

Data will be made available on request.

## Notes

Composition of experimental diets; identity and structural classification of lignin-derived pyrolysis products; amount of water-unextractable solids and analyzed lignin contents; fecal lignin content and structure per individual pig; lignin intake and output during the 2 day total feces collection period; mean apparent digestibility of nutrients; ratio marker and nutrient in digesta and feed; semiquantification of indole pyrolysis product; SEC profile of wheat straw and fecal lignin isolates; wheat straw lignin contents analyzed with conventional markers in the sample matrix (PDF)
