# Supplementary material for: Lignin in Fibrous Feed as an Internal Digestibility and Transit Marker in Pigs
Source: ACS Agric Sci Technol. 2025 Apr 24;5(6):1025–33. doi: 10.1021/acsagscitech.4c00752 (PMC12175119; doi:10.1021/acsagscitech.4c00752)
Supplement: Supplementary file 1 [file as4c00752_si_001.pdf]

Supporting information for

Lignin in fibrous feed as internal digestibility and transit marker in pigs

Romy J. Veersma[a], Corentin Lannuzel[b], Walter J.J. Gerrits[b], Sonja de Vries[b], Gijs van Erven[a,c]

Mirjam A. Kabel[a,]*

[a]Laboratory of Food Chemistry, Wageningen University & Research, Bornse Weilanden 9, 6708 WG, Wageningen, The Netherlands

[b]Animal Nutrition Group, Wageningen University & Research, De Elst 1, 6708 WS, Wageningen, The Netherlands

[c]Wageningen Food & Biobased Research, Wageningen University & Research, Bornse Weilanden 9, 6708 WG, Wageningen, The Netherlands

*Corresponding author:

Dr. ir. Mirjam A. Kabel.

Email: mirjam.kabel@wur.nl

Address: Bornse Weilanden 9, 6708 WG, Wageningen, The Netherlands

Supplementary material contents:

Tables:

Table S1: Ingredients and analysed composition of experimental wheat straw (WS)-based diets, without (a; prior to total collection of faeces) or with (b; prior to dissection) external markers. Composition was analysed in (a) diets excluding TiO_2, Co-EDTA and Cr-mordanted WS, and assumed to be similar in diets including these external markers. Adapted from Lannuzel et al. (2024).[1]

Table S2: Identity and structural classification of lignin-derived pyrolysis products by ^{13}C-IS pyrolysis-GC-MS.[3]

Table S3: Amount of water-unextractable solids (WUS), prepared in singlicate according to section 2.3, and corresponding lignin contents (% w/w, WUS dry matter) as determined in triplicate with ^{13}C-IS py-GC-MS. Means ± standard deviation of analytical triplicates.

Table S4: Lignin content (% w/w, dry matter) of FEC_{WSC} per pig, as determined with ^{13}C-IS py-GC-MS. Lignin was analysed in WUS and calculated back to total sample (Table S3). Corresponding lignin recoveries (%) from the diet to faeces were calculated based on lignin input and output (Table S6). ^{13}C-IS py-GC-MS relative abundances of lignin compounds in FEC_{WSC} were corrected for relative response factors and relative abundance of ^{13}C analogues. Sum based on structural classification according Table S2. Means ± standard deviation of analytical triplicates.

Table S5: Lignin content (% w/w, dry matter) of FEC_{WSF} per pig, as determined with ^{13}C-IS py-GC-MS. Lignin was analysed in WUS and calculated back to total sample (Table S3). Corresponding lignin recoveries (%) from the diet to faeces were calculated based on lignin input and output (Table S6). ^{13}C-IS py-GC-MS relative abundances of lignin compounds in FEC_{WSF} were corrected for relative response factors and relative abundance of ^{13}C analogues. Sum based on structural classification according Table S2. Means ± standard deviation of analytical triplicates.

Table S6: Feed, wheat straw and lignin intake (g, dry matter (DM)) and faecal and lignin output (g, DM) during the 2-day total faeces collection period. Means ± standard deviation of biological replicates (n=6).

Table S7: Mean apparent digestibility (% of intake)[a] of dry matter (DM), nitrogen (N) and fat in pigs (n=12) fed diets containing 135 g/kg wheat straw, supplied either as coarse (WSC) or fine (WSF), calculated using either intrinsic lignin or titanium (originating from TiO_2) as marker. Mean apparent digestibility values are determined from rectal (n=12) and ileal (n=8) digestibility estimates.

Table S8: $Lignin_{digesta}/Lignin_{feed}$, $Ti_{digesta}/Ti_{feed}$, ratio between $Lignin_{digesta}/Lignin_{feed}$ and $Ti_{digesta}/Ti_{feed}$, and $Nutrient_{digesta}/Nutrient_{feed}$ for dry matter (DM), nitrogen (N) and fat. The values in bold present the discussed two outliers in Fig 2, section 3.2. Means ± standard deviation of biological replicates (n=6).

Figures:

Fig S1: Indole pyrolysis product (area/ µg sample), dry matter based, of water-unextractable solids (WUS) of wheat straw coarse (WSC), wheat straw fine (WSF) and corresponding faeces (n=6).

Fig S2: Alkaline SEC elution profile of mild organosolv lignins isolated from wheat straw coarse (WSC_{LIG}) and corresponding pooled faeces ($FEC_{P\text{-}WSC\text{-}LIG}$). ● = feedstock, ● = faeces

Fig S3: Lignin content (% w/w) in non-extracted WSC, as determined with ^{13}C-IS py-GC-MS, with titanium dioxide (Ti) or sodium dichromate dihydrate (Cr) mixed into to the sample matrix. Means ± standard deviation of analytical triplicates. Note that concentrations of Ti and Cr in rectal and ileal samples did not exceed 1.5% (w/w). Concentrations tested here exceed this value.

Table S1: Ingredients and analysed composition of experimental wheat straw (WS)-based diets, without (a; prior to total collection of faeces) or with (b; prior to dissection) external markers. Composition was analysed in (a) diets excluding TiO_2, Co-EDTA and Cr-mordanted WS, and assumed to be similar in diets including these external markers. Adapted from Lannuzel et al. (2024).[1]

	Content (g/kg, as is)			
**Ingredient**	**WSC (a)**	**WSC (b)**	**WSF (a)**	**WSF (b)**
WS (≤ 20 mm)	150.0	135.0	-	-
WS (≤ 1 mm)	-	-	150.0	135.0
Maize starch (native)	483.0	473.0	483.0	473.0
Potato protein	40.0	40.0	40.0	40.0
Casein	40.0	40.0	40.0	40.0
Fish meal[a]	92.0	92.0	92.0	92.0
Soy oil	58.0	58.0	58.0	58.0
Sugar	100.0	100.0	100.0	100.0
Premix[b]	5.0	5.0	5.0	5.0
Magnesium sulfide	5.0	5.0	5.0	5.0
Limestone	10.0	10.0	10.0	10.0
Monocalcium phosphate	5.0	5.0	5.0	5.0
Potassium carbonate	4.0	4.0	4.0	4.0
Salt	1.0	1.0	1.0	1.0
Sodium bicarbonate	5.0	5.0	5.0	5.0
DL-Methionine	1.0	1.0	1.0	1.0
L-tryptophan	1.0	1.0	1.0	1.0
TiO_2	-	4.0	-	4.0
Co-EDTA	-	1.0	-	1.0
Polyethylene glycol 8000 (PEG)	-	5.0	-	5.0
Cr-mordanted WS (≤ 20 mm)	-	15.0	-	-
Cr-mordanted WS (≤ 1 mm)	-	-	-	15.0
	**Phytase unit (FTU)**			
Phytase	500.0	500.0	500.0	500.0
**Analysed composition**	**Content (g/kg, as is)**			
Dry matter	921.8	-	923.3	-
Crude protein (N x 6.25)	139.3	-	140.9	-
Crude fat	66.2	-	66.5	-
Starch	389.3	-	391.4	-
Non-starch polysaccharides[c]	85.8	-	83.4	-
Arabinosyl	4.1	-	4.1	-
Xylosyl	30.1	-	30.7	-
Mannosyl	0.6	-	0.6	-
Galactosyl	0.8	-	1.0	-
Glucosyl	51.8	-	50.0	-
Uronyl	4.0	-	4.2	-
Ash	65.7	-	66.3	-
	**MJ ME/kg (as is)**			

Metabolisable energy[d]	15.0	15.0

[a]70% crude protein; [b]Premix composition (x/kg diet): 10000 IU vitamin A, 2000 IU vitamin D_3, 40 mg vitamin E, 1.5 mg vitamin K_3, 1.0 mg vitamin B_1, 4.0 mg vitamin B_2, 1.5 mg vitamin B_6, 20 µg vitamin B_{12}, 30 mg niacin, 15 mg D-pantothenic acid, 150 mg choline chloride, 0.4 mg folic acid, 0.05 mg Biotin; 100 mg Fe (as $FeSO_4.H_2O$), 20 mg Cu (as $CuSO_4.5H_2O$), 30 mg Mn (as MnO), 70 mg Zn (as $ZnSO_4.H_2O$), 0.70 mg I (as KI), 0.25 mg Se (as Na_2SeO_3). Carrier: milled maize; [c]Presented as anhydrosugars; [d]ME = ((20.0 * digestible crude protein) + (39.1 * digestible ether extract) + (17.5 * starch) + (16.6 * sugars) + (17.2 * digestible non starch polysaccharides))/ 1000.[2]

Table S2: Identity and structural classification of lignin-derived pyrolysis products by ^{13}C-IS pyrolysis-GC-MS.[3]

**Compound**	**Sub-unit**[a]	**Structural feature**[b]	**Side chain length**	**Mw**[c] **^{12}C**	**Quan ion ^{12}C [M-*e*]**	**Mw**[c] **^{13}C**	**Quan ion ^{13}C [M-*e*]**
phenol	H	unsub	0	94	94.04132	100	100.06145
guaiacol	G	unsub	0	124	124.05188	131	115.04853
2-methylphenol	H	methyl	Cα	108	108.05698	115	115.08045
4-methylphenol/ 3-MP	H	methyl	Cα	108	107.04914	115	114.07263
4-methylguaiacol	G	methyl	Cα	138	138.06753	146	146.09437
2,4-dimethylphenol	H	methyl	Cα	122	107.04914	130	114.07263
4-ethylphenol	H	misc	Cβ	122	107.04914	130	114.07263
4-ethylguaiacol	G	misc	Cβ	152	137.05971	161	145.08654
4-vinylguaiacol	G	vinyl	Cβ	150	150.06753	159	159.09754
4-vinylphenol	H	vinyl	Cβ	120	120.05697	128	128.08380
eugenol	G	misc	Cɣ	164	164.08318	174	174.11673
4-propylguaiacol	G	misc	Cɣ	166	137.05971	175	145.08654
syringol	S	unsub	0	154	154.06245	162	162.08928
cis-isoeugenol	G	misc	Cɣ	164	164.08318	174	174.11673
4-propenylphenol	H	misc	Cɣ	134	133.06479	143	142.09498
trans-isoeugenol	G	misc	Cɣ	164	164.08318	174	174.11673
4-methylsyringol	S	methyl	Cα	168	168.07810	177	177.10829
vanillin	G	Cα-O	Cα	152	151.03897	160	159.06581
4-propyneguaiacol	G	misc	Cɣ	162	162.06753	172	172.10108
4-alleneguaiacol	G	misc	Cɣ	162	162.06753	172	172.10108
homovanillin	G	Cβ-O	Cβ	166	137.05971	175	145.08654
4-ethylsyringol	S	misc	Cβ	182	167.07022	192	176.10046
vanillic acid methyl ester	G	Cα-O	Cα	182	182.05736	191	191.08766
acetovanillone	G	Cα-O	Cβ	166	151.03897	175	159.06581
4-hydroxybenzaldehyde	H	Cα-O	Cα	122	121.02848	129	128.05189
4-vinylsyringol	S	vinyl	Cβ	180	180.07810	190	190.11164
guaiacylacetone	G	Cβ-O	Cɣ	180	137.05971	190	145.08654
4-allylsyringol	S	misc	Cɣ	194	194.09373	205	205.13065
propiovanillone	S	Cα-O	Cɣ	180	151.03897	190	159.06581
guaiacyl vinyl ketone	G	Cα-O	Cɣ	178	151.03897	188	159.06581

guaiacyl diketone	G	Cα-O, Cβ-O	Cɣ	194	151.03897	204	159.06581
cis-4-propenylsyringol	S	misc	Cɣ	194	194.09375	205	205.13065
4-propynesyringol	S	misc	Cɣ	192	192.07810	203	203.11500
4-allenesyringol	S	misc	Cɣ	192	192.07810	203	203.11500
trans-4-propenylsyringol	S	misc	Cɣ	194	194.09375	205	205.13065
dihydroconiferyl alcohol	G	Cɣ-O	Cɣ	182	137.05971	192	145.08654
syringaldehyde	S	Cα-O	Cα	182	182.05736	191	191.08755
cis-coniferyl alcohol	G	Cɣ-O	Cɣ	180	137.05971	190	145.08654
cis-coumaryl alcohol	H	Cɣ-O	Cɣ	150	107.04921	159	114.07269
homosyringaldehyde	S	Cβ-O	Cβ	196	167.07027	206	176.10046
syringic acid methyl ester	S	Cα-O	Cα	212	212.06793	222	222.10147
acetosyringone	S	Cα-O	Cβ	196	181.04948	206	190.07973
trans-coumaryl alcohol	H	Cɣ-O	Cɣ	150	107.04921	159	114.07268
trans-coniferyl alcohol	G	Cɣ-O	Cɣ	180	137.05971	190	145.08654
trans-coniferaldehyde	G	Cɣ-O	Cɣ	178	147.04406	188	156.07425
syringylacetone	S	Cβ-O	Cɣ	210	167.07027	221	176.10046
propiosyringone	S	Cα-O	Cɣ	210	181.04954	221	190.07973
syringyl diketone	S	Cα-O, Cβ-O	Cɣ	224	181.04954	235	190.07973
syringyl vinylketone	S	Cα-O	Cɣ	208	181.04954	219	190.07973
dihydrosinapyl alcohol	S	Cɣ-O	Cɣ	212	168.07841	223	177.10829
cis-sinapyl alcohol	S	Cɣ-O	Cɣ	210	167.07027	221	176.10046
trans-sinapyl alcohol	S	Cɣ-O	Cɣ	210	167.07027	221	176.10046
trans-sinapaldehyde	S	Cɣ-O	Cɣ	208	208.07301	219	219.10994

[a]*p*-hydroxyphenyl (H) unit, guaiacyl (G) unit, syringyl (S) unit.; [b]Structural features: unsubstituted (unsub), methyl group, miscellaneous (misc), vinyl group, Cα-oxidised (Cα-O), Cβ-oxidised (Cβ-O), Cɣ-oxidised (Cɣ-O); [c]Molecular weight (M_w) in g/mol.

Table S3: Amount of water-unextractable solids (WUS), prepared in singlicate according to section 2.3, and corresponding lignin contents (% w/w, WUS dry matter) as determined in triplicate with ^{13}C-IS py-GC-MS. Means ± standard deviation of analytical triplicates.

Sample	Pig nr.	% WUS	Lignin content (% w/w, WUS)
**WSC**	-	91.8	19.8 ± 0.4
**WSF**	-	90.8	19.2 ± 0.4
**FEC_{WSC}**	1	82.9	16.3 ± 0.9
	2	83.1	16.9 ± 0.5
	3	85.1	16.9 ± 0.1
	4	83.1	15.8 ± 0.7
	5	82.8	15.6 ± 0.4
	6	84.1	16.7 ± 0.6
**FEC_{WSF}**	7	86.0	16.3 ± 0.9
	8	86.5	16.3 ± 0.6
	9	85.8	16.4 ± 0.4
	10	85.2	17.1 ± 1.3
	11	86.3	17.2 ± 0.7
	12	87.3	18.0 ± 0.7

Table S4: Lignin content (% w/w, dry matter) of FEC_{WSC} per pig, as determined with ^{13}C-IS py-GC-MS. Lignin was analysed in WUS and calculated back to total sample (Table S3). Corresponding lignin recoveries (%) from the diet to faeces were calculated based on lignin input and output (Table S6). ^{13}C-IS py-GC-MS relative abundances of lignin compounds in FEC_{WSC} were corrected for relative response factors and relative abundance of ^{13}C analogues. Sum based on structural classification according Table S2. Means ± standard deviation of analytical triplicates.

	**FEC_{WSC} Pig 1**	**FEC_{WSC} Pig 2**	**FEC_{WSC} Pig 3**	**FEC_{WSC} Pig 19**	**FEC_{WSC} Pig 20**	**FEC_{WSC} Pig 21**
**Lignin content (% w/w)**	13.5 ± 0.8	14.0 ± 0.4	14.3 ± 0.1	13.2 ± 0.6	13.0 ± 0.3	14.0 ± 0.5
**Lignin recovery (diet to faeces) (%)**	67	60	70	73	67	76

**Lignin subunits (%)**						
H	13.5 ± 0.3	13.6 ± 0.8	13.8 ± 0.2	14.3 ± 1.0	14.6 ± 0.7	14.7 ± 0.2
G	59.9 ± 0.3	59.1 ± 0.8	60.1 ± 0.4	60.0 ± 0.6	58.9 ± 1.2	59.5 ± 1.0
S	26.6 ± 0.3	27.3 ± 0.6	26.2 ± 0.6	25.7 ± 1.4	26.5 ± 1.0	25.8 ± 1.2
S/G	0.44 ± 0.0	0.46 ± 0.0	0.44 ± 0.0	0.43 ± 0.0	0.45 ± 0.0	0.43 ± 0.0
**Coumaryl-, coniferyl and sinapyl alcohol (%)**						
CouA[a]	3.0 ± 0.2	3.1 ± 0.0	3.2 ± 0.1	3.1 ± 0.2	3.0 ± 0.1	3.0 ± 0.1
ConA[b]	58.5 ± 0.9	58.2 ± 0.9	59.0 ± 0.6	58.4 ± 0.6	57.3 ± 1.2	57.4 ± 0.7
SinA[c]	38.5 ± 0.8	38.7 ± 0.8	37.8 ± 0.5	38.5 ± 0.7	39.7 ± 1.3	39.6 ± 0.8
SinA/ConA	0.66 ± 0.0	0.67 ± 0.0	0.64 ± 0.0	0.66 ± 0.0	0.69 ± 0.0	0.69 ± 0.0
**Lignin structural moieties (%)[d]**						
Unsubstituted	6.7 ± 0.3	6.8 ± 0.1	6.7 ± 0.3	6.9 ± 0.1	7.2 ± 0.2	7.2 ± 0.2
Methyl	4.3 ± 0.2	4.3 ± 0.0	4.3 ± 0.3	4.4 ± 0.1	4.3 ± 0.1	4.2 ± 0.2
Vinyl	41.8 ± 0.5	41.0 ± 0.9	42.0 ± 1.0	43.6 ± 2.2	43.4 ± 0.1	45.1 ± 1.2
4-VP[e]	9.6 ± 0.2	9.8 ± 0.7	9.9 ± 0.2	10.3 ± 0.8	10.3 ± 0.6	10.7 ± 0.2
4-VG[f]	28.0 ± 0.4	26.9 ± 0.5	28.1 ± 0.8	29.2 ± 1.4	28.8 ± 0.6	30.1 ± 1.2
Cα-ox	4.5 ± 0.1	4.3 ± 0.2	4.3 ± 0.1	4.3 ± 0.3	4.4 ± 0.1	4.2 ± 0.1
Cβ-ox	1.1 ± 0.0	1.1 ± 0.0	1.1 ± 0.0	1.1 ± 0.1	1.1 ± 0.0	1.1 ± 0.0
Cγ-ox	36.6 ± 0.7	37.2 ± 0.8	36.3 ± 1.6	34.7 ± 2.1	34.8 ± 0.2	33.2 ± 1.0
Miscellaneous	5.0 ± 0.2	5.3 ± 0.0	5.2 ± 0.7	5.1 ± 0.2	4.8 ± 0.1	5.2 ± 0.1
PhCγ[g]	42.7 ± 0.7	43.5 ± 0.8	42.5 ± 1.0	40.7 ± 2.1	40.6 ± 0.1	39.3 ± 1.1
PhCγ-corrected[h]	42.0 ± 0.7	42.8 ± 0.8	41.8 ± 1.0	40.0 ± 2.0	39.9 ± 0.1	38.7 ± 1.1

[a]*t*-coumaryl alcohol; [b]*t*-coniferyl alcohol; [c]*t*-sinapyl alcohol; [d]Some lignin-derived pyrolysis products can be indicative for more than one structural moiety, hence the total exceeds 100%; [e]4-vinylphenol; [f]4-vinylguaiacol; [g]phenols with intact α,β,γ carbon side chain; [h]phenols with intact α,β,γ carbon side chain, excluding diketones and vinylketones.

Table S5: Lignin content (% w/w, dry matter) of FEC_{WSF} per pig, as determined with ^{13}C-IS py-GC-MS. Lignin was analysed in WUS and calculated back to total sample (Table S3). Corresponding lignin recoveries (%) from the diet to faeces were calculated based on lignin input and output (Table S6). ^{13}C-IS py-GC-MS relative abundances of lignin compounds in FEC_{WSF} were corrected for relative response factors and relative abundance of ^{13}C analogues. Sum based on structural classification according Table S2. Means ± standard deviation of analytical triplicates.

	**FEC_{WSF} Pig 7**	**FEC_{WSF} Pig 8**	**FEC_{WSF} Pig 9**	**FEC_{WSF} Pig 22**	**FEC_{WSF} Pig 23**	**FEC_{WSF} Pig 24**
**Lignin content (% w/w)**	14.0 ± 0.8	14.1 ± 0.5	14.1 ± 0.3	14.6 ± 1.1	14.9 ± 0.6	15.8 ± 0.6
**Lignin recovery (diet to faeces) (%)**	51	77	66	84	77	81

**Lignin subunits (%)**						
H	14.2 ± 0.1	14.4 ± 0.7	14.3 ± 0.5	14.3 ± 0.4	13.9 ± 0.9	14.2 ± 0.3
G	60.0 ± 0.3	59.0 ± 1.1	60.5 ± 0.8	59.7 ± 0.9	58.8 ± 0.2	59.1 ± 0.7
S	25.8 ± 0.3	26.5 ± 1.7	25.2 ± 0.3	26.0 ± 1.0	27.2 ± 1.0	26.7 ± 0.9
S/G	0.43 ± 0.0	0.45 ± 0.0	0.42 ± 0.0	0.44 ± 0.0	0.46 ± 0.0	0.45 ± 0.0
**Coumaryl-, coniferyl and sinapyl alcohol (%)**						
CouA[a]	2.9 ± 0.1	2.9 ± 0.0	3.0 ± 0.1	2.9 ± 0.0	2.8 ± 0.2	3.0 ± 0.2
ConA[b]	57.4 ± 0.4	56.2 ± 0.8	58.2 ± 0.4	57.0 ± 1.0	56.1 ± 0.7	56.8 ± 0.4
SinA[c]	39.7 ± 0.3	40.9 ± 0.8	38.8 ± 0.4	40.1 ± 1.0	41.1 ± 0.6	40.2 ± 0.6
SinA/ConA	0.69 ± 0.0	0.73 ± 0.0	0.67 ± 0.0	0.70 ± 0.0	0.73 ± 0.0	0.71 ± 0.0
**Lignin structural moieties (%)[d]**						
Unsubstituted	6.7 ± 0.2	6.7 ± 0.1	6.8 ± 0.2	6.8 ± 0.0	6.7 ± 0.1	6.6 ± 0.2
Methyl	4.3 ± 0.3	4.0 ± 0.3	4.5 ± 0.2	4.2 ± 0.3	3.9 ± 0.2	4.3 ± 0.1
Vinyl	45.2 ± 0.5	44.6 ± 2.1	44.6 ± 0.3	44.8 ± 1.3	43.0 ± 1.8	44.2 ± 1.2
4-VP[e]	10.4 ± 0.2	10.7 ± 0.7	10.2 ± 0.4	10.4 ± 0.4	10.2 ± 0.8	10.5 ± 0.3
4-VG[f]	30.5 ± 0.3	29.6 ± 1.7	30.2 ± 0.7	30.2 ± 1.1	28.6 ± 1.0	29.3 ± 1.1
Cα-ox	4.3 ± 0.2	4.2 ± 0.1	4.0 ± 0.0	4.2 ± 0.1	4.4 ± 0.1	4.3 ± 0.1
Cβ-ox	1.1 ± 0.0	1.1 ± 0.1	1.0 ± 0.0	1.1 ± 0.1	1.1 ± 0.0	1.1 ± 0.0
Cγ-ox	33.3 ± 1.0	34.5 ± 2.4	34.1 ± 0.5	33.7 ± 1.6	36.2 ± 1.9	34.0 ± 1.4
Miscellaneous	5.1 ± 0.3	4.9 ± 0.4	5.0 ± 0.2	5.1 ± 0.3	4.6 ± 0.2	5.5 ± 0.1
PhCγ[g]	39.5 ± 0.7	40.5 ± 2.2	39.9 ± 0.2	39.8 ± 1.5	41.9 ± 1.8	40.5 ± 1.3
PhCγ-corrected[h]	38.8 ± 0.7	39.8 ± 2.2	39.3 ± 0.3	39.1 ± 1.4	41.2 ± 1.8	39.8 ± 1.3

[a]*t*-coumaryl alcohol; [b]*t*-coniferyl alcohol; [c]*t*-sinapyl alcohol; [d]Some lignin-derived pyrolysis products can be indicative for more than one structural moiety, hence the total exceeds 100%; [e]4-vinylphenol; [f]4-vinylguaiacol; [g]phenols with intact α,β,γ carbon side chain; [h]phenols with intact α,β,γ carbon side chain, excluding diketones and vinylketones.

Table S6: Feed, wheat straw and lignin intake (g, dry matter (DM)) and faecal and lignin output (g, DM) during the 2-day total faeces collection period. Means ± standard deviation of biological replicates (n=6).

Diet	Pig	Feed intake (g, DM)	Straw intake (g, DM)	Lignin intake (g, DM)	Faecal output (g, DM)	Lignin output (g, DM)
**WSC**	1	3207	479	87	433	58
	2	3232	481	87	375	53
	3	3343	499	91	441	63
	4	3199	477	87	480	63
	5	3199	477	87	446	58
	6	3155	470	85	465	65
	Mean	3222 ± 64	481 ± 10	87 ± 2	440 ± 36	60 ± 5
**WSF**	7	3035	457	80	287	40
	8	3253	490	86	464	65
	9	3375	509	89	418	59
	10	3271	494	86	498	73
	11	3430	516	90	467	69
	12	2983	449	78	401	63
	Mean	3224 ± 180	486 ± 27	85 ± 5	422 ± 75	62 ± 12

Table S7: Mean apparent digestibility (% of intake)[a] of dry matter (DM), nitrogen (N) and fat in pigs (n=12) fed diets containing 135 g/kg wheat straw, supplied either as coarse (WSC) or fine (WSF), calculated using either intrinsic lignin or titanium (originating from TiO_2) as marker. Mean apparent digestibility values are determined from rectal (n=12) and ileal (n=8) digestibility estimates.

	**Nutrient**		
	**DM**	**N**	**Fat**
**Lignin**	77.7* ± 0.59	82.6 ± 1.18	93.3 ± 0.54
**Titanium**	74.1* ± 0.95	80.2 ± 0.91	92.5 ± 0.56
**Model *P*-value**[b]	<0.0001	<0.0001	<0.0001

[a]Data are presented as Least Squares Means (LSMeans) and relative standard deviation (%; calculated as pooled SDx100/|LSMean|); [b]Model established *P*-values for the fixed effect of method, segment and diet. There were no diet interactions (diet x method, diet x segment, and diet x method x segment; $P > 0.3$), accordingly these interactions were omitted from the model.*Digestibility values with an asterisk differ significantly at $P < 0.05$ between marker methods.

Table S8: $\text{Lignin}_{digesta}/\text{Lignin}_{feed}$, $\text{Ti}_{digesta}/\text{Ti}_{feed}$, ratio between $\text{Lignin}_{digesta}/\text{Lignin}_{feed}$ and $\text{Ti}_{digesta}/\text{Ti}_{feed}$, and $\text{Nutrient}_{digesta}/\text{Nutrient}_{feed}$ for dry matter (DM), nitrogen (N) and fat. The values in bold present the discussed two outliers in Fig 2, section 3.2. Means ± standard deviation of biological replicates (n=6).

**Diet**	**Pig**	**Segment**	**$\text{Lignin}_{digesta}/\text{Lignin}_{feed}$**	**$\text{Ti}_{digesta}/\text{Ti}_{feed}$**	**Ratio**	**$\text{DM}_{digesta}/\text{DM}_{feed}$**	**$\text{N}_{digesta}/\text{N}_{feed}$**	**$\text{Fat}_{digesta}/\text{Fat}_{feed}$**
**WSC**	2	Ileum	3.2	4.3	0.7	1.0	1.0	0.3
	**3**		**4.5**	**2.7**	**1.7**	**1.0**	**0.7**	**0.2**
	**4**		**5.1**	**2.5**	**2.0**	**1.0**	**0.5**	**0.2**
	5		3.5	3.4	1.0	1.0	1.1	0.3
	1	Rectum	5.1	3.8	1.1	1.0	0.9	0.2
	2		5.2	4.0	1.2	1.0	0.9	0.2
	3		5.5	3.0	1.4	1.0	0.9	0.2
	4		4.8	4.1	1.1	1.0	0.7	0.2
	5		5.1	5.0	1.0	1.0	0.7	0.5
	6		4.6	4.8	1.1	1.1	0.8	0.5
**WSF**	7	Ileum	4.2	3.8	1.1	1.0	0.9	0.2
	8		4.6	4.0	1.2	1.0	0.9	0.2
	10		4.0	3.0	1.4	1.0	0.9	0.2
	11		4.4	4.1	1.1	1.0	0.7	0.2
	7	Rectum	5.2	5.0	1.0	1.0	0.7	0.5
	8		5.8	4.8	1.1	1.1	0.8	0.5
	9		5.9	6.0	0.9	1.1	0.7	0.5
	10		5.6	3.9	1.2	1.0	0.6	0.4
	11		5.3	4.0	1.3	1.0	0.9	0.5
	12		5.4	4.7	1.0	1.0	0.6	0.5
	Mean	Ileum & Rectum	4.9 ± 0.7	4.4 ± 1.0	1.2 ± 0.3	1.0 ± 0.0	0.8 ± 0.1	0.3 ± 0.1

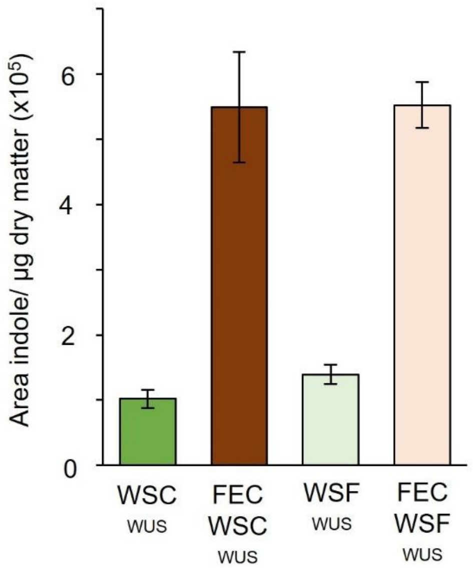

Fig S1: Indole pyrolysis product (area/ µg sample), dry matter based, of water-unextractable solids (WUS) of wheat straw coarse (WSC), wheat straw fine (WSF) and corresponding faeces (n=6).

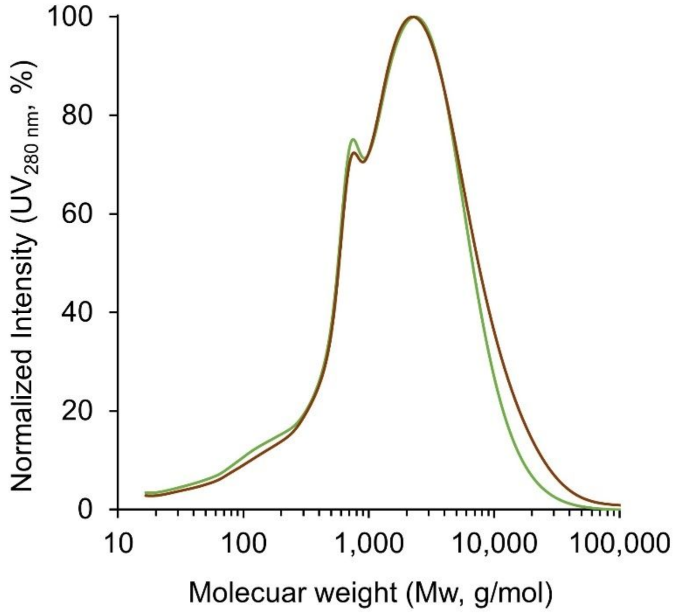

Fig S2: Alkaline SEC elution profile of mild organosolv lignins isolated from wheat straw coarse (WSC_{LIG}) and corresponding pooled faeces ($FEC_{P\text{-}WSC\text{-}LIG}$). ● = feedstock, ● = faeces

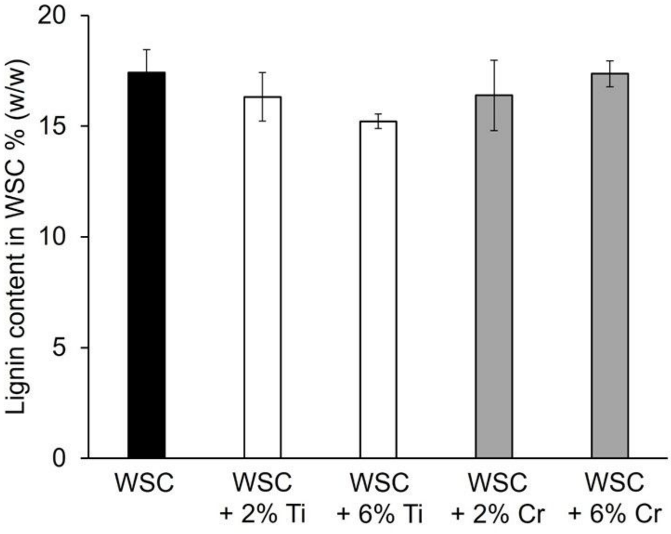

Fig S3: Lignin content (% w/w) in non-extracted WSC, as determined with ^{13}C-IS py-GC-MS, with titanium dioxide (Ti) or sodium dichromate dihydrate (Cr) mixed into to the sample matrix. Means ± standard deviation of analytical triplicates. Note that concentrations of Ti and Cr in rectal and ileal samples did not exceed 1.5% (w/w). Concentrations tested here exceed this value.

References

(1) Lannuzel, C.; Veersma, R. J.; Wever, N.; van Erven, G.; Kabel, M. A.; Gerrits, W. J. J.; de Vries, S., Particle Size of Insoluble Fibres and Gelation of Soluble Fbres Influence Digesta Passage Rate Throughout the Gastrointestinal Tract of Finishing Pigs. *animal* **2024**, 101175. DOI: 10.1016/j.animal.2024.101175.

(2) Noblet, J.; Fortune, H.; Shi, X. S.; Dubois, S., Prediction of Net Energy Value of Feeds for Growing Pigs. *J. Anim. Sci.* **1994**, *72* (2), 344-54. DOI: 10.2527/1994.722344x.

(3) Veersma, R. J.; van Erven, G.; Lannuzel, C.; de Vries, S.; Kabel, M. A., Quantitative ^{13}C-IS Pyrolysis-GC-MS Lignin Analysis: Overcoming Matrix Effects in Animal Feed and Faeces, *J. Anal. Appl. Pyrolysis*, **2024**, 183, 106802. DOI: 10.1016/j.jaap.2024.106802.
